# The role of school performance in narrowing gender gaps in the formation of STEM aspirations: a cross-national study

**DOI:** 10.3389/fpsyg.2015.00171

**Published:** 2015-02-25

**Authors:** Allison Mann, Joscha Legewie, Thomas A. DiPrete

**Affiliations:** ^1^Department of Sociology, Columbia UniversityNew York, NY, USA; ^2^Department of Humanities and Social Sciences, New York UniversityNew York, NY, USA

**Keywords:** education, school context, gender inequality, careers in science, technology, engineering, mathematics, cross-cultural research

## Abstract

This study uses cross-national evidence to estimate the effect of school peer performance on the size of the gender gap in the formation of STEM career aspirations. We argue that STEM aspirations are influenced not only by gender stereotyping in the national culture but also by the performance of peers in the local school environment. Our analyses are based on the Program for International Student Assessment (PISA). They investigate whether 15-year-old students from 55 different countries expect to have STEM jobs at the age of 30. We find considerable gender differences in the plans to pursue careers in STEM occupations in all countries. Using PISA test scores in math and science aggregated at the school level as a measure of school performance, we find that stronger performance environments have a negative impact on student career aspirations in STEM. Although girls are less likely than boys to aspire to STEM occupations, even when they have comparable abilities, boys respond more than girls to competitive school performance environments. As a consequence, the aspirations gender gap narrows for high-performing students in stronger performance environments. We show that those effects are larger in countries that do not sort students into different educational tracks.

## 1. Introduction

A growing body of research documents the under-representation of women in science, technology, engineering, and mathematics (STEM) occupations and fields of study (Xie and Shauman, 2003; Eccles, 2007; Ceci and Williams, 2011; Ceci et al., 2014). In order to understand the sources of these differences, we need to study the formation of career aspirations in high school, because high school aspirations are strong predictors of initial college major choice and the attainment of a Bachelor degree in STEM fields (Tai et al., 2006; Morgan et al., 2013; Legewie and DiPrete, 2014a). Recent research from the United States demonstrates that the high school environment—and particularly the strength of the STEM curriculum and the gender segregation of extra-curricula activities—have a substantial impact on gender differences in plans to major in STEM fields in college (Legewie and DiPrete, 2014b). Data from South Korea suggest that single-sex schools for boys increase the level of interest in STEM fields, but single-sex schools for girls do not have a corresponding effect on the STEM aspirations of girls (Park et al., 2012). Related research finds that the school context also plays an important role for gender differences in educational performance (Legewie and DiPrete, 2012).

In this study, we contribute to research on the role of the school context for the gender gap in STEM aspirations, by examining the impact that peer ability has on gender differences in the formation of STEM orientations across 55 countries. Researchers have found that the school performance environment has a negative impact on student career aspirations in science (Marsh and Hau, 2003; Shen and Tam, 2008; Nagengast and Marsh, 2012). There is strong theoretical justification for expecting a gender difference in the responsiveness to the school performance climate. High performance in the environment arguably raises the level of competition. It has important implications for the self evaluation of performance, which in turn shapes the aspirations for different fields of study. Indeed, the self evaluation of performance plays a central role in previous research. With respect to women and STEM fields, (Correll, 2001) argues that gender status beliefs lead boys to evaluate their math and science abilities more highly than girls do, either because girls believe that the relative competency assessment is valid or because girls expect that others will accept the ranking as valid. Correll (2001) found that the undervaluation by girls of their own competence in math had behavioral consequences in that it discouraged them from pursuing quantitative coursework and fields of study. Other researchers have reached similar conclusions with regard to the influence of self evaluations on course choices in high school (Marsh and Yeung, 1997; Nagy et al., 2008) and career aspirations (Eccles et al., 1999; Nagy et al., 2006; Riegle-Crumb et al., 2010; Eccles, 2011; Sikora and Pokropek, 2012).

Similar to gender status beliefs that influence performance expectations, the ability of peers in the school context provides an important reference for performance evaluations and an important influence on the formation of STEM field aspirations. This influence is presumably twofold. First, peer ability influences the self-evaluation of math and science ability and aspirations for STEM fields directly. Second, peer ability mediates the role of performance for self-evaluation and aspirations insofar as the influence of performance on aspirations (returns to performance) varies depending on the performance of peers. Previous research on the country level supports this idea. Mann and DiPrete (unpublished manuscript) show that boys and girls have lower STEM aspirations and stronger returns to math-science performance when they live in countries with stronger overall performance levels. This finding is attributed to the higher risk of failure in more competitive environments and the concomitant need for stronger evidence that one is good at math-science before forming a STEM orientation. Mann and DiPrete (unpublished manuscript) also find that the effect on the math-science slope of a stronger math-science country environment is stronger for girls than for boys, which is linked to gender status beliefs in the national culture. If this is true, we would expect to find a similar pattern in school environments, particularly during the high school years.

The influence of peer ability most likely differs across countries. We examine these variations in a sample of 55 countries and point to the importance of tracking systems as a mediating factor for peer influence on STEM aspirations. The track into which a student is placed affects the composition of the student's peer group and provides an independent signal of the student's ability and potential. The organization of national education systems has been shown to influence student's educational aspirations in previous studies. Research shows that in relatively undifferentiated (unstructured) systems—where there are fewer tracks and a later age at first selection into tracks—peer and parent attitudes have significantly greater influences on student aspirations to complete college and to pursue high-status occupations (Buchmann and Dalton, 2002; Buchmann and Park, 2009). Furthermore, students in course tracking appear to experience the opposite patterns: although lower self assessments typically emerge in higher-performance environments, students in higher tracks have higher self assessments (Chmielewski et al., 2013). Environmental and contextual factors also have been shown to influence academic self assessments and career intentions aside from the aggregate impact of school performance or SES (Alwin and Otto, 1977; Legewie and DiPrete, 2014b). Accordingly, structural features of national and school education systems might influence the extent to which peer ability shapes educational aspirations.

## 2. Data and methods

Measures and sample data are from the Program for International Student Assessment (PISA). PISA is a triennial international study that tests the reading, mathematical and scientific literacy level of 15-year-old students who are still in school. The database is hierarchically structured such that students are nested within schools, and schools are nested within countries. We use the 2006 data collection, which included 57 countries. In 2006, science was the major content domain.

We restrict our sample in three ways. First, we exclude data from Qatar because the students were not asked about STEM aspirations. Second, we exclude students in schools that have fewer than 10 students considering that we are interested in understanding school effects (about 6800 observations). Finally, we remove data from Liechtenstein because of the small number of schools (about 12 schools and 300 observations). With these restrictions, there are 55 countries, 12,846 schools, and 331,834 students in the final sample.

### 2.1. Dependent variable: STEM aspirations

The dependent variable is whether the student expects to have a STEM job at the age of 30. The question taken from the student questionnaire was “What kind of job do you expect to have when you are about 30 years old? Write the job title **–**.” The responses were coded using the International Standard Classification of Occupations. Our definition excludes some of the occupations that have been treated as STEM occupations in previous research (Kjærnsli and Lie, 2011; Sikora and Pokropek, 2012)—specifically, nursing and associate or technician level occupations—because we are interested in a measure of aspirations for STEM careers among high-performing students. In some models, we use the STEM subfields of physical sciences and life sciences as the dependent variables (always relative to those with non-STEM aspirations). The Appendix includes a detailed list of occupations for STEM fields and the breakdown between the physical and life sciences.

### 2.2. Math and science performance

PISA does not contain information about student grades or other performance feedback given directly to students. We use test scores—the best measure of performance—as a proxy for all observed and unobserved performance feedback available to students. The composite math and science test scores for each student were averaged to form an individual math-science test score. We standardized the average of the math-science test scores for the students in each country; within each country the math-science test score measure has a mean of zero and a standard deviation of 1. Then, we aggregated the standardized test score measure to the school level to create a measure of the school performance environment. With these measures, we are able to identify the high- and low-performing students and schools in each country, but we obscure the relative position of students in the global sample.

### 2.3. Demographic characteristics

We use demographic information about each respondent—specifically sex, immigrant status, a broad measure of socio-economic status (ESCS)—and an indicator for whether either parent has a science-related career. PISA respondents are all 15 years old so that age is not a relevant predictor, but we do include the student's grade level relative to the modal grade for the country in which the student lives.

### 2.4. Country characteristics

We use measures of the structural features of the nation's education system as they pertain to the tracking of students between schools. We use a binary measure for whether assignment into tracks occurs before the age of 16. Countries with an early age at first selection into tracks are also countries that tend to have more programs in which 15-year old students are enrolled. Thus, as an alternative measure of national tracking, we use the number of separate programs in which 15-year old students can be enrolled (a binary variable that measures whether this number is greater than one). Because these variables represent the same underlying concept, they are not used in the same models.

### 2.5. Procedures

To determine whether the school context is related to the gender gap in STEM aspirations, we use regression analyses with country fixed effects and standard errors clustered on schools. We use logistic regression predicting three different dependent variables—STEM aspirations, physical science aspirations, and life science aspirations. The dependent variable was regressed onto standardized test scores, standardized school performance measures and their interaction, and gender. In addition, we include gender interactions with all performance measures and also with the background measures described above. To assess cross-national variation in the magnitude of these effects, we use hierarchical logistic regression models.

## 3. Results

This section begins with descriptions of the sample countries in terms of our variable of interest – STEM-related aspirations. Table 1 shows the results of the descriptive statistics—both overall and by gender. Because PISA has a complex, two-stage stratified sample design, all descriptive statistics are weighted using the student-level weights provided in the dataset to compensate for unequal selection probabilities of students.

**Table 1:** **Nation-level descriptive statistics**.

**Variable**	**Obs**	**Mean**	**Std. Dev**.	**Min**.	**Max**.	**Male mean**	**Female mean**
Own math-science (MS)	55	0	1		0.064	−0.060	
School math-science (SchMS)	55	0	0.6	0.3	0.8	−0.014	0.013
ESCS	55	−0.175	0.487	−1.434	0.823	−0.145	−0.202
Immigrant	55	0.092	0.126	0.001	0.734	0.092	0.092
Parent STEM career	55	0.072	0.030	0.007	0.139	0.074	0.070
Relative grade level	55	−0.131	0.302	−0.947	0.553	−0.167	−0.010
STEM aspirations	55	0.219	0.080	0.087	0.469	0.239	0.199
Physical science aspirations	55	0.121	0.050	0.045	0.292	0.175	0.069
Life science aspirations	55	0.127	0.067	0.046	0.344	0.097	0.151
First age of selection into tracks	54	14.176	1.901	10	17		
Number of programs	54	2.315	1.226	1	5		
No ability grouping	54	0.341	0.206	0.003	0.895	0.340	0.342
Ability grouping-some classes	54	0.456	0.243	0.033	0.918	0.455	0.458
Ability grouping-all classes	54	0.203	0.176	0.007	0.770	0.205	0.201

Across the 55 countries, the average proportion of students with STEM aspirations is 22 percent, ranging from a low of about 9 percent in Montenegro to a high of about 47 percent in Colombia. The average proportion of students with life science aspirations is about 12 percent, as is the average proportion of students with physical science aspirations. These proportions mask significant variability; some countries—Mexico, Chile, Brazil, and Colombia—have 25 percent of students or more with life science aspirations, and other countries—Switzerland, the Netherlands, Austria, and Germany—have only 5–6 percent of students with life science aspirations. The Latin American countries also have large proportions of students with physical sciences aspirations, while several European and Asian countries have very low proportions of students with physical science aspirations compared with the global average.

In most countries, we observe substantial gender differences in STEM aspirations. There is a male advantage in physical science aspirations in 52 of 55 countries (with no significant gender difference in 3 countries). There is a female advantage in life science aspirations in 48 countries, a male advantage in 1 country, and no significant gender difference in life science aspirations in 6 countries. With all STEM occupations combined (referred to as “combined STEM” below), males have an advantage in STEM aspirations in 34 countries in the study, females have an advantage in 6 countries, and there is no significant gender difference in STEM aspirations in the remaining countries. The magnitude of the gender gap in STEM aspirations varies considerably, with a 10-point difference in proportions favoring girls in Kyrgyzstan and a 16-point difference in proportions favoring boys in Chinese Taipei.

### 3.1. Analytical results

We begin our analysis of gender differences in STEM aspirations by pooling the students across countries and estimating three logistic regression models that predict overall STEM aspirations, physical science aspirations, and life science aspirations. Because we are interested in the average effects of school performance environments, these models use country fixed effects to condition on all observed and unobserved factors on the country level. These models use cluster robust standard errors to account for clustering on schools. Table 2 displays the results.

**Table 2:** **Gender differences in the effects of the local performance environment on STEM aspirations, with country fixed effects**.

	**Overall STEM**		**Physical sciences**		**Life sciences**	
	**Coef**	**S. E**.	**Coef**	**S. E**.	**Coef**	**S. E**.
Female	−0.23^***^	0.01	−1.14^***^	0.02	0.60^***^	0.02
Math-science score (MS)	0.65^***^	0.01	0.66^***^	0.01	0.64^***^	0.01
ESCS	0.09^***^	0.01	0.05^***^	0.01	0.12^***^	0.01
Immigrant	0.49^***^	0.02	0.41^***^	0.03	0.55^***^	0.03
Parent in STEM Occup.	0.47^***^	0.02	0.43^***^	0.02	0.51^***^	0.02
Relative grade level	−0.04^***^	0.01	−0.00	0.01	−0.07^***^	0.01
School math-science (SchMS)	−0.03^*^	0.01	−0.04^**^	0.02	−0.02	0.03
**INTERACTIONS**						
SchMS × MS	−0.10^***^	0.02	−0.11^***^	0.01	−0.10^***^	0.02
Female × MS	−0.10^***^	0.01	−0.01	0.02	−0.11^***^	0.02
Female × SchMS	−0.11^***^	0.02	−0.10^**^	0.03	−0.13^***^	0.03
Female × MS × SchMS	0.12^***^	0.02	0.10^***^	0.02	0.10^***^	0.02
Constant	−1.29^***^	0.07	−2.38^***^	0.11	−2.05^***^	0.08
Number of observations	322947		284663		285972	

* *p < 0.05*;

** *p < 0.01*;

*** *p < 0.001*.

Generally speaking, girls respond differently to the school performance environment than do boys. Strong environments decrease only slightly the propensity for boys to develop STEM aspirations at the mean of the individual-level performance distribution. However, the negative interaction between own performance and school performance means that strong performance environments more powerfully suppress STEM aspirations for stronger-performing boys (*p* < 0.001). The negative interaction between school environment and female means that the gender gap in physical science aspirations widens in favor of boys in stronger school environments for students at the mean of the math-science distribution (*p* < 0.01), while the female advantage in life science aspirations shrinks in stronger school environments (*p* < 0.001). At the same time, however, in the aspirations model, the three-way interaction between school environment, own performance, and female is significantly positive (Female × MS × SchMS = 0.12, *p* < 0.001). This means that the widening gender gap in high-performance schools applies more to weaker performing students than stronger performing students. Boys have a tendency to “de-differentiate” by own performance in stronger environments. Girls show no such tendency; their tendency to differentiate by own performance when forming STEM aspirations remains as strong in high performance environments as in low performance environments. This pattern applies both to physical science and to life science STEM aspirations. As a result, the remaining analysis focuses on gender differences in the response to performance environments for combined-STEM aspirations.

**Figure 1:**
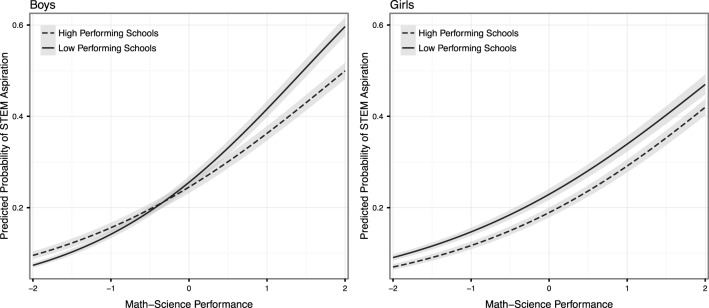
**Predicted probabilities of STEM aspirations for boys and girls in different school environments across the math-science distribution**.

To further illustrate the gender differences in the response to school performance environments for STEM aspirations, Figure 1 plots the predicted probabilities of having a STEM aspiration across the math-science distribution for boys and girls in schools at the 10th percentile (“low performing schools,” SchMS = −0.74) and at the 90th percentile (“high performing schools,” SchMS = 0.89) of the distribution of school math-science environments. Table 3 contains the corresponding predicted probabilities of a high-, average- and low-performing student in high- and low-performing schools. Figure 1 (and all subsequent figures) assume the “base case” (i.e., setting all independent variables to zero), which corresponds at a substantive level to a native-born student in the modal grade for the country, with average socio-economic status, parents in non-STEM occupations, and average values on test-score measures except as otherwise indicated. As Figure 1 shows, girls have lower STEM aspirations than boys in most circumstances, but girls have an advantage relative to boys in the difference between the returns to math-science in strong performance environments and in low performance environments. This is because boys receive higher returns to math-science scores in lower performance environments than they do in higher performance environments while girls receive similar returns without regard to the strength of the school performance environment. To put it another way, the gender gap in STEM aspirations among high performing students is smaller when these students are in higher performance environments. High performance school environments provide lower costs to girls than they do to boys.

**Table 3:** **Predicted probabilities of STEM aspirations for boys and girls in different school environments and at different positions on the math-science distribution**.

	**High-performing schools**		**Low-performing schools**	
	**Boys**	**Girls**	**Boys**	**Girls**
High-performing student	0.38	0.31	0.44	0.35
Average-performing student	0.23	0.18	0.24	0.22
Low-performing student	0.13	0.10	0.12	0.13

*High-performing and low-performing schools are defined as schools in the 90th and 10th percentiles of the school MS distribution, respectively. High-, average-, and low-performing students are defined as students at the 90th, 50th, and 10th percentiles of the MS distribution, respectively*.

### 3.2. Country differences in the importance of school performance for gender differences in STEM aspirations

It is important to keep in mind that these results are averages across all the PISA countries and themselves mask potentially strong environmental heterogeneity. Having established the average importance of school performance environments for STEM aspirations and gender differences in the response to school performance environments, we therefore next use hierarchical models to examine heterogeneity across countries in the effects of school environments on STEM aspirations. We estimate separate models for boys and girls that use STEM aspirations as the dependent variable. Each model includes own math-science, school math-science, and their interaction, as the predictor variables, as well as controls for socio-economic status, immigrant status, having a parent with a STEM occupation, and relative grade level. Each model includes random intercepts at the country and school level and random country slopes for own math-science, school math-science, and their interaction. In these models, the “fixed” effects are consistent with the output shown in Table 2 (see also the first set of models in **Table 5**).

**Figure 2:**
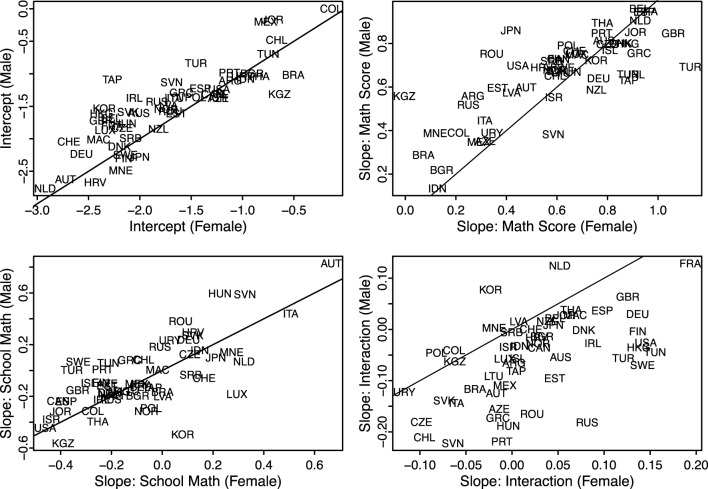
**Country random-effects estimates from models predicting STEM aspirations for boys and girls**.

Table 4 contains the total effects for each country (including the random components). Figure 2 displays these results graphically by presenting the male effect on the y-axis and the female effect on the x-axis, with a 45° reference line representing gender parity, for each of the four estimates of interest. As expected, the regression intercepts are larger for boys (i.e., above the 45° line) in most but not all country environments. The returns to math-science test scores are positive in all countries and are stronger for boys (i.e., above the 45° line) in most country environments. The returns to school performance environments are negative in the majority of countries, but there is a sizable minority of countries where the returns to school performance environments are positive. Many of the countries with large positive coefficients for SchMS (Austria, Croatia, Germany, Italy, Montenegro, Slovenia, and the Slovak Republic) have structural features of their national education system that facilitate tracking into homogeneous environments. Gender differences in the interaction of own math-science times school math-science (MS×SchMS) favor girls in the majority of countries. This corresponds to the finding from the country fixed-effects models (Table 2) that the female response to own math-science performance is greater (depending on the country, increases more or decreases less) than is the male response in schools with stronger math-science environments.

**Table 4:** **Total effects of performance and performance environment on STEM aspirations, by gender**.

	**Intercept**		**Math-science slope**		**School M-S slope**		**Interaction**	
	**Girls**	**Boys**	**Girls**	**Boys**	**Girls**	**Boys**	**Girls**	**Boys**
**COUNTRIES WITH EARLY TRACKING (BEFORE AGE 16)**								
Argentina	−1.119	−1.102	0.267	0.560	−0.172	−0.161	0.002	−0.065
Austria	−2.725	−2.625	0.477	0.598	0.665	0.828	−0.017	−0.125
Azerbaijan	−1.236	−1.374	0.316	0.352	−0.217	−0.100	−0.014	−0.155
Belgium	−2.280	−1.683	0.926	0.960	−0.087	−0.127	0.047	0.022
Bulgaria	−0.906	−0.995	0.144	0.219	−0.097	−0.195	0.033	−0.013
Chile	−0.667	−0.478	0.592	0.652	−0.074	0.084	−0.095	−0.211
Chinese Taipei	−2.268	−1.087	0.883	0.631	−0.039	−0.132	0.005	−0.081
Colombia	−0.136	0.003	0.210	0.390	−0.274	−0.314	−0.063	−0.041
Croatia	−2.434	−2.670	0.539	0.690	0.122	0.296	0.027	−0.016
Czech Republic	−2.172	−1.832	0.798	0.797	0.109	0.126	−0.098	−0.181
Estonia	−1.645	−1.609	0.365	0.597	−0.217	−0.117	0.046	−0.095
France	−2.270	−1.803	0.956	0.948	−0.082	−0.114	0.194	0.129
Germany	−2.569	−2.233	0.764	0.640	0.104	0.237	0.136	0.030
Greece	−1.595	−1.290	0.925	0.756	−0.128	0.080	−0.015	−0.173
Hong Kong-China	−2.372	−1.609	0.879	0.799	−0.198	−0.194	0.137	−0.035
Hungary	−2.147	−1.765	0.650	0.669	0.227	0.595	−0.004	−0.188
Indonesia	−0.971	−1.079	0.127	0.134	0.148	0.157	0.008	−0.032
Ireland	−2.054	−1.370	0.914	0.658	−0.239	−0.225	0.088	−0.027
Israel	−1.237	−1.363	0.587	0.553	−0.438	−0.378	−0.004	−0.034
Italy	−1.663	−1.370	0.314	0.446	0.506	0.440	−0.060	−0.144
Japan	−2.002	−2.280	0.418	0.860	0.211	0.099	0.045	0.009
Korea	−2.346	−1.532	0.755	0.723	0.081	−0.495	−0.023	0.078
Kyrgyzstan	−0.640	−1.312	−0.004	0.558	−0.391	−0.563	−0.062	−0.062
Lithuania	−1.664	−1.369	0.615	0.666	−0.128	−0.160	−0.020	−0.091
Luxembourg	−2.337	−1.868	0.678	0.752	0.294	−0.183	−0.008	−0.057
Macao-China	−2.402	−2.011	0.679	0.748	−0.018	0.006	0.069	0.028
Mexico	−0.773	−0.198	0.291	0.348	−0.101	−0.102	−0.008	−0.109
Montenegro	−2.185	−2.486	0.118	0.388	0.273	0.142	−0.019	0.003
Netherlands	−2.923	−2.764	0.930	0.905	0.322	0.070	0.052	0.124
Portugal	−1.127	−0.983	0.777	0.848	−0.235	0.009	−0.011	−0.219
Romania	−1.703	−1.575	0.342	0.753	0.072	0.382	0.022	−0.165
Russian Federation	−1.834	−1.428	0.249	0.519	−0.009	0.184	0.082	−0.181
Serbia	−2.091	−1.989	0.578	0.720	0.112	−0.033	0.000	−0.005
Slovak Republic	−2.115	−1.586	0.599	0.674	0.119	0.272	−0.074	−0.139
Slovenia	−1.690	−1.130	0.585	0.383	0.326	0.590	−0.064	−0.222
Switzerland	−2.691	−2.043	0.669	0.766	0.165	−0.058	0.021	0.000
Turkey	−1.453	−0.837	1.127	0.692	−0.356	0.003	0.121	−0.056
Uruguay	−1.003	−1.037	0.343	0.390	0.030	0.234	−0.117	−0.122
**COUNTRIES WITHOUT EARLY TRACKING**								
Australia	−2.007	−1.603	0.785	0.813	−0.203	−0.223	0.053	−0.054
Brazil	−0.505	−1.016	0.072	0.288	0.001	−0.166	−0.040	−0.116
Canada	−1.291	−1.315	0.605	0.728	−0.409	−0.236	0.030	−0.036
Denmark	−2.199	−2.118	0.852	0.802	−0.210	−0.168	0.078	−0.001
Finland	−2.157	−2.302	0.597	0.730	−0.241	−0.093	0.137	−0.004
Iceland	−1.215	−1.303	0.809	0.769	−0.289	−0.096	0.006	−0.056
Jordan	−0.705	−0.165	0.911	0.853	−0.401	−0.316	0.057	0.028
Latvia	−1.719	−1.489	0.420	0.574	0.002	−0.200	0.007	0.016
New Zealand	−1.819	−1.848	0.756	0.588	−0.212	−0.181	0.038	0.016
Norway	−1.745	−1.542	0.591	0.678	−0.063	−0.315	0.028	−0.027
Poland	−1.457	−1.362	0.643	0.791	−0.044	−0.291	−0.082	−0.046
Spain	−1.411	−1.225	0.944	0.945	−0.378	−0.239	0.098	0.037
Sweden	−2.141	−2.246	0.622	0.690	−0.331	0.068	0.142	−0.070
Thailand	−0.841	−1.037	0.780	0.894	−0.254	−0.395	0.064	0.038
Tunisia	−0.750	−0.695	0.881	0.658	−0.212	0.058	0.155	−0.044
United Kingdom	−2.378	−1.720	1.060	0.847	−0.333	−0.152	0.126	0.064
United States	−1.229	−1.237	0.445	0.699	−0.461	−0.444	0.145	−0.026

**Figure 3:**
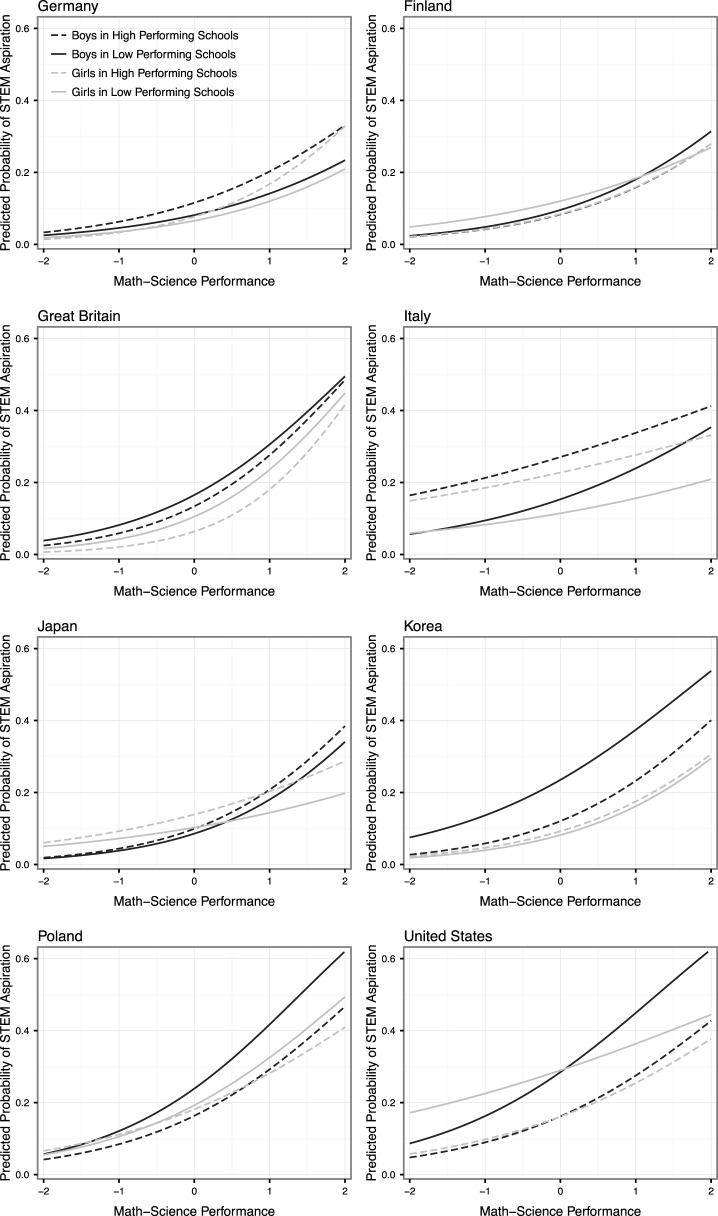
**Predicted probabilities of STEM aspirations for boys and girls in different school environments across the math-science distribution (selected countries)**.

As Figure 2 (bottom right panel) shows, however, this pattern—while widely present—is not universal. While most countries are below the 45° line, a few countries are above it. Figure 3 displays the predicted probabilities of having a STEM aspiration across the math-science distribution for boys and girls in schools at the 10th and 90th percentile of the school math-science distribution in 8 selected countries that show nation-level variability in the relative effect of school environments on STEM aspirations for boys and for girls. In Italy and Korea, girls have lower STEM aspirations than boys in the base case (MS = 0, SchMS = 0), but the relative difference in aspirations is smaller in higher-performing schools; conversely, in Japan girls have higher aspirations than boys in the base case, and the aspirations gap widens in higher-performing schools. In all three of these countries, girls have higher STEM aspirations in higher-performing schools than they do in lower-performing schools. In Italy and Japan, boys have higher math-science test score slopes. However, in Italy the male math-science slopes are smaller in higher-performing schools—that is, own performance has a bigger effect on STEM aspirations for boys in low-performing schools—but girls' slopes do not respond to the school environment. Conversely, in Japan, boys' math-science slopes respond very little to the school environment, but girls' slopes increase in stronger performance environments. In Korea, girls have slightly higher returns to math-science, but those slopes do not change in the school environment, while the male slopes increase in high-performing environments^1^.

School environments affect STEM aspirations differently in the remaining 5 countries, which are examples of the dominant pattern found in the PISA data in the bottom right panel of Figure 2. In Finland, girls have higher STEM aspirations at average ability levels (MS = 0), especially when they are in low-performing schools; boys' math-science slopes are larger, but those returns diminish in stronger performance environments. In the United States, boys and girls have comparable aspirations at average ability levels (MS = 0) without regard to school environments, but (similar to Finland) boys have larger math-science slopes, with diminishing gender differences in effects in stronger performance environments (where girls' slopes converge across school performance levels and boys' slopes diverge). In Poland and Great Britain, girls have lower STEM aspirations in the base case; however in Great Britain, the effects widen in stronger performance environments, while in Poland, they narrow. Similarly, girls' math-science slopes are larger than boys' slopes in Great Britain, while the reverse is true in Poland. In both cases, those gender differences are heightened in stronger performance environments.

To explore whether structural features of country and school education systems explain the variation in the effects of the performance environment on gender differences in STEM aspirations, we estimated a similar set of models on subsets of the data selected according to the characteristics of the school systems. Table 5 presents the estimates from the random-effects models predicting STEM aspirations. The first set of models for boys and for girls use the full sample. The second set of models use the subset of countries that have no between-school tracking before age 16. The final set of models use the subset of countries that have between-school tracking before age 16. Looking across the columns, the most noticeable difference is the effects on students of average ability levels (MS = 0) of the school performance environment. In countries without tracking, STEM aspirations significantly decrease in stronger performance environments, which is consistent with a social comparison effect, while in countries with tracking, STEM aspirations significantly increase in stronger performance environments, which is consistent with a signal associated with placement in a higher track. Strong environments in the absence of a signal about a student's track placement appears to weaken student intentions to pursue a STEM career. But in the presence of a signal about track placement, strong environments (which invariably means placement in an academic track) enhance student intentions to pursue a STEM career. In models predicting science self assessments (available upon request), the relative pattern of the school performance slopes is similar; the school performance effects on self assessments are more strongly negative in less structured school environments than in environments with national tracking systems. This suggests that part of the mechanism for the effect of the performance environment on STEM aspirations runs through science self assessments^2^.

**Table 5:** **Gender differences in the effects of the local performance environment on STEM aspirations, with country and school random effects**.

	**Full sample**				**No tracking before age 16**				**Tracking before age 16**			
	**Girls**		**Boys**		**Girls**		**Boys**		**Girls**		**Boys**	
	**Coef**	**S. E**.	**Coef**	**S. E**.	**Coef**	**S. E**.	**Coef**	**S. E**.	**Coef**	**S. E**.	**Coef**	**S. E**.
Math-science score (MS)	0.62	0.04	0.67	0.03	0.65	0.05	0.69	0.04	0.56	0.06	0.63	0.04
ESCS	0.09	0.01	0.10	0.01	0.11	0.01	0.13	0.01	0.08	0.01	0.06	0.01
Immigrant	0.52	0.03	0.57	0.03	0.57	0.03	0.65	0.03	0.39	0.05	0.39	0.05
Parent in STEM Occup.	0.36	0.02	0.54	0.02	0.36	0.03	0.47	0.03	0.36	0.04	0.63	0.04
Relative grade level	−0.10	0.01	0.01	0.01	−0.13	0.02	0.02	0.02	−0.08	0.02	−0.01	0.02
School math-science (SchMS)	−0.06	0.04	−0.03	0.05	−0.26	0.04	−0.21	0.04	0.19	0.06	0.20	0.08
SchM × MS	0.03	0.02	−0.05	0.02	0.05	0.02	−0.06	0.02	−0.01	0.03	−0.06	0.03
Intercept	−1.75	0.09	−1.54	0.08	−1.52	0.11	−1.39	0.10	−2.00	0.13	−1.70	0.14
**Error terms**	**Std. Dev**.		**Std. Dev**.		**Std. Dev**.		**Std. Dev**.		**Std. Dev**.		**Std. Dev**.	
School intercept	0.51		0.50		0.44		0.43		0.61		0.58	
Country intercept	0.67		0.61		0.61		0.53		0.65		0.68	
Country MS slope	0.29		0.20		0.29		0.20		0.27		0.20	
Country SchMS slope	0.28		0.31		0.17		0.15		0.24		0.34	
Country MS × SchMS slope	0.10		0.11		0.06		0.07		0.08		0.12	
Number of observations	169457		154754		92678		82168		74867		69710	

**Figure 4:**
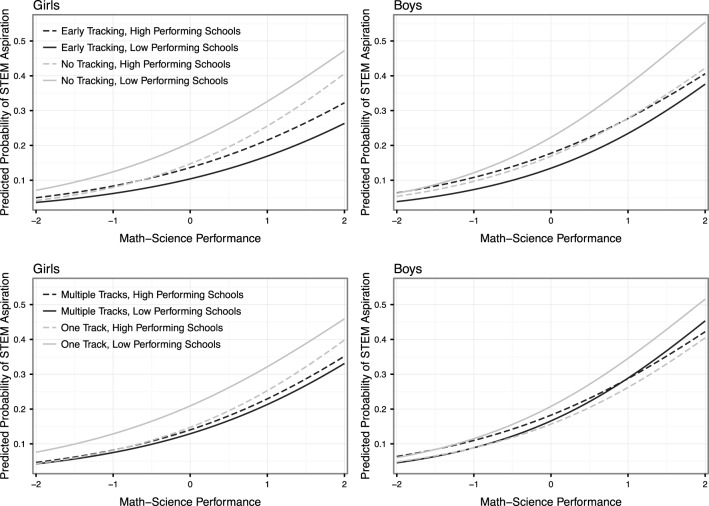
**Predicted probabilities of STEM aspirations for boys and girls in different school environments across the math-science distribution and by characteristics of national tracking system**.

The relative difference across genders in the interaction effect of own math-science and school math-science also is greater in countries that do not track students between schools. Figure 4 displays the results of models estimated separately for students in different types of national education systems; the top two panels show the results for girls and boys in countries that begin tracking students into schools before the age of 16 compared to those that do not begin tracking students into schools before age 16, and the bottom two panels show the results for girls and boys in countries that have multiple tracks into which students are assigned compared to those students in countries where only one track is possible. In general, students in low-performing schools (the solid lines in Figure 4) who live in countries with institutional tracking receive the lowest returns to math-science performance. Our interpretation is that the signal given to these students by their track placement crowds out the signal they are receiving from their own math-science performance in these countries. In the countries without tracking, on the other hand, the effect of the own-performance signal is relatively strong. The own-performance signal is especially strong in low-performing schools. In high performing untracked schools, the social comparison effect of high performing peers reduces the probability of STEM aspirations for high performing students. Figure 4 also shows in the top two panels that the gender gap in STEM aspirations in favor of boys is diminished in untracked schools when these schools are high performance schools^3^.

## 4. Discussion

This paper examines the impact that peer ability has on gender differences in the formation of STEM orientations. Peer ability is measured by a school's math and science performance level. High performance in the environment arguably raises the level of competition. Across the 55 countries in our sample, we show that girls and boys are more likely to develop STEM orientations if they have stronger performance in math and science; yet in high performance school environments, boys and girls require stronger evidence that they are good in math and science before deciding to pursue a STEM orientation. This is consistent with the pattern for nation-level performance and STEM aspirations (Mann and DiPrete, unpublished manuscript). In general, however, strong environments have different effects for girls and for boys. Strong environments generally widen the gender gap in physical science aspirations in favor of boys and shrink the female advantage in life science aspirations, but—as Table 2 makes clear—this impact primarily falls on low performing students. Among high performing students, stronger math-science environments shrinks the overall STEM gender gap. These patterns are not universal, however. Countries display heterogeneity in the effects of the school performance environment on STEM aspirations and in particular the impact of the performance environment on student decision-making in response to their own level of math-science performance.

Some of this country variation can be attributed to country differences in the structure of tracking. Our analysis made clear that the strength of the own-performance signal on STEM aspirations is stronger in countries that do not use early tracking in their school systems than in countries with early tracking. In early tracking school systems, STEM aspirations are generally higher in the high performing schools (the “academic” track). In untracked school systems, STEM aspirations are generally higher at any given level of own performance in low-performing schools, and this gap in favor of low-performing schools grows as own performance increases. We see this as clear evidence of a social comparison effect in strong performance environments. Moreover, there is a clear gender difference in the workings of this social comparison effect. Boys respond more strongly to their own performance than do girls in environments that provide weak signals from tracking and in environments where peer performance is weak, which seems to induce strongly performing boys more than girls to draw the conclusion that they belong in STEM occupations. In environments with strong environmental performance, the gender gap in STEM aspirations shrinks. In other words, girls who perform well in environments filled with other strong performing students behave more similarly to boys in the formation of their STEM aspirations. Again, however, there is country-heterogeneity in the responses to own performance and environmental signals that our models cannot fully account for.

### Conflict of interest statement

The authors declare that the research was conducted in the absence of any commercial or financial relationships that could be construed as a potential conflict of interest.

## Footnotes

^1^Recall that Korea is one of the few countries in the sample in which boys have a larger slope on the interaction of individual math-science and school performance than girls.

^2^We use the science self-concept scale as a measure of self assessment. Because our models reveal no significant gender differences in the effects of peer performance on self assessments or in the returns to own math-science for self assessments, we do not report them here.

^3^To further explore the sources of cross-national variation in effects of performance and performance environments, we included the country Gender Gap Index (2006) in the separate models for boys and girls in tracking and non-tracking subsets of the sample. The inclusion of the GGI had no significant effect on the estimates of interest. The GGI lowers STEM aspirations to a comparatively greater extent for girls than for boys, and it reduces the variability in the country regression intercept.
