# Supplementary material for: The role of school performance in narrowing gender gaps in the formation of STEM aspirations: a cross-national study
Source: Front Psychol. 2015 Feb 25;6:171. doi: 10.3389/fpsyg.2015.00171 (PMC4340185; doi:10.3389/fpsyg.2015.00171)
Supplement: Supplementary file 1 [file DataSheet1.PDF]

Appendix A STEM Occupations

Physical Sciences

1236 Computing services department managers

1237 Research and development department managers

2100 Physical, mathematical

2100 Physical, mathematical, and engineering science professionals

2110 Physicists, chemists, and related professionals

2111 Physicists and astronomers

2112 Meteorologists

2113 Chemists

2114 Geologists and geophysicists including geodesists

2120 Mathematicians and statisticians

2121 Mathematicians and associated professionals

2122 Statisticians including actuaries

2130 Computing professionals

2131 Computer systems designers and analysts including software engineers

2132 Computer programmers

2139 Computing professionals not elsewhere classified

2140 Architects, engineers and related professionals

2141 Architects, town and traffic planners including landscape architects

2142 Civil engineers including construction engineers

2143 Electrical engineers

2144 Electronics and telecommunications engineers

2145 Mechanical engineers

2146 Chemical engineers

2147 Mining engineers, metallurgists and related professionals

2148 Cartographers and surveyors

2149 Architects engineers and related professionals not elsewhere classified

Life Sciences

2200 Life science and health professionals

2210 Life science professionals

2211 Biologists, botanists, and zoologists

2212 Pharmacologists, pathologists, and biochemists

2213 Agronomists

2220 Health professionals (except nursing)

2221 Medical doctors

2222 Dentists

2223 Veterinarians

2224 Pharmacists

2229 Health professionals except nursing not elsewhere classified
